# Supplementary material for: Ultrasonographic characteristics of major salivary glands in anti-centromere antibody-positive primary Sjögren’s syndrome
Source: PLoS One. 2021 Nov 3;16(11):e0259519. doi: 10.1371/journal.pone.0259519 (PMC8565722; doi:10.1371/journal.pone.0259519)
Supplement: S1 Table — (DOCX) [file pone.0259519.s001.docx]

**Supplementary Table 1. Salivary gland ultrasonography scores of patients with ACA positive and age-, and USFR- propensity score matched ACA negative primary Sjogren’s syndrome**

|  | Anti-centromere antibody positive pSS, (N=19) | Anti-centromere antibody negative pSS, (N=38)* | *P* |
| --- | --- | --- | --- |
| Hypoechoic area score of parotid gland (0-6) | 2.0 [ 1.5; 2.5] | 3.5 [ 1.0; 4.0] | 0.035 |
| Hypoechoic area score of submandibular gland (0-6) | 2.0 [ 1.0; 3.0] | 4.0 [ 2.0; 4.5] | 0.117 |
| Total hypoehoic area score (0-12) | 4.0 [ 3.5; 5.0] | 8.0 [ 3.0; 8.0] | 0.045 |
| Hyperechoic foci score of parotid gland (0-6) | 2.0 [ 2.0; 2.0] | 2.0 [ 2.0; 2.5] | 0.163 |
| Hyperechoic foci score of submandibular gland (0-2) | 2.0 [ 2.0; 2.0] | 2.0 [ 2.0; 2.0] | 0.630 |
| Total hyperechoic foci score (0-8) | 4.0 [ 3.5; 4.0] | 4.0 [ 3.5; 4.0] | 0.159 |
| Echogenicity score of parotid gland (0-2) | 0.0 [ 0.0; 1.0] | 0.0 [ 0.0; 2.0] | 0.386 |
| Echogenicity score of submandibular gland (0-2) | 0.0 [ 0.0; 2.0] | 1.0 [ 0.0; 2.0] | 0.433 |
| Total echogenicity score (0-4) | 0.5 [ 0.0; 2.0] | 2.0 [ 0.0; 4.0] | 0.346 |
| Homogeneity score of parotid gland (0-6) | 2.0 [ 2.0; 4.0] | 2.0 [ 1.0; 4.5] | 0.871 |
| Homogeneity score of submandibular gland (0-6) | 3.5 [ 2.0; 5.0] | 4.0 [ 2.0; 5.0] | 0.806 |
| Total homogeneity score (0-12) | 6.0 [ 4.0; 8.0] | 6.0 [ 3.0;10.0] | 0.946 |
| Clearance of the border score of parotid gland (0-6) | 0.0 [ 0.0; 0.0] | 0.0 [ 0.0; 2.0] | 0.230 |
| Clearance of the border score of submandibular gland (0-6) | 2.0 [ 0.0; 3.0] | 2.0 [ 0.0; 3.0] | 0.479 |
| Total clearance of the border score (0-12) | 2.0 [ 0.0; 4.0] | 2.0 [ 0.0; 3.5] | 0.816 |
| PDUS score of parotid gland (0-6) | 1.5 [ 0.0; 3.0] | 1.0 [ 0.0; 4.0] | 0.497 |
| PDUS score of submandibular gland (0-6) | 1.0 [ 0.0; 4.0] | 1.0 [ 0.0; 3.0] | 1.000 |
| Total PDUS score (0-12) | 2.5 [ 0.0; 6.0] | 3.5 [ 0.0; 6.0] | 0.765 |
| SGUS score of parotid gland, (0-26) | 7.0 [ 4.0; 10.0] | 10.0 [ 3.0;13.5] | 0.070 |
| SGUS score of submandibular gland, (0-22) | 9.0 [ 5.5;12.0] | 13.0 [ 5.0;14.5] | 0.031 |
| Total SGUS score, (0-48) | 16.0 [ 11.5;21.5] | 21.5 [ 9.0;28.0] | 0.046 |
| SGUS score ≥ 14, N (%) | 12 (63.2%) | 26 (68.4%) | 0.432 |

PDUS, power Doppler ultrasonography; SGUS, salivary gland ultrasonography

Continuous variables are presented as mean ± standard deviation or median with interquartile range depending on whether it is normally distributed or not.

* Age-, and USFR- matched ACA negative primary Sjogren’s syndrome group was selected by propensity score matching with nearest-neighboring and 1:2 ratio.
